# Railway Car Sanitation Bill

**Published:** 1903-04

**Authors:** 


### RAILWAY CAR SANITATION BILL.

(Caption and enacting clause omitted.)

"'Section 1. It shall be the duty of the State Health Officer of Texas, and he is hereby authorized and empowered to prepare rules and regulations governing the proper disinfection and sanitation of public buildings and all railway coaches, and sleeping cars operated in the State of Texas.'

"'Sec. 2. It shall be his duty, and he is hereby authorized and empowered to prescribe a sanitary code which shall contain and provide rules and regulations of a general nature for the improvement and amelioration of the hygienic and sanitary condition of said public buildings, railway coaches and sleeping cars.'

" 'Sec. 3. Every person having control of any public building, railway company, sleeping car company, or other corporation, company or individual or the receiver thereof, engaged in the conveying of passengers in this State shall, at their own expense, within a prescribed time after receiving notice from the State Health Officer of the promulgation of the rules and regulations in the above sections mentioned, carry the same into effect.'

" 'Sec. 4. If any person having control of any public building or any agent, manager, operator, employe, or receiver of any railway company, sleeping car company, or any individual, shall fail to comply with the provisions of this act, and the rules and regulations promulgated by the State Health Officer under the provisions hereof, he shall be deemed guilty of a misdemeanor, and upon conviction shall be punished by a fine of not less than fifty nor more than two hundred dollars.' "

Emergency clause stricken out. Effect in ninety days.

